# A New Ligand-Based Method for Purifying Active Human Plasma-Derived Ficolin-3 Complexes Supports the Phenomenon of Crosstalk between Pattern-Recognition Molecules and Immunoglobulins

**DOI:** 10.1371/journal.pone.0156691

**Published:** 2016-05-27

**Authors:** Aleksandra Man-Kupisinska, Mateusz Michalski, Anna Maciejewska, Anna S. Swierzko, Maciej Cedzynski, Czeslaw Lugowski, Jolanta Lukasiewicz

**Affiliations:** 1 Department of Immunochemistry, Hirszfeld Institute of Immunology and Experimental Therapy, Polish Academy of Sciences, Wroclaw, Poland; 2 Laboratory of Immunobiology of Infections, Institute of Medical Biology, Polish Academy of Sciences, Lodz, Poland; 3 Institute of Microbiology, Immunology and Biotechnology, University of Lodz, Lodz, Poland; University of Leicester, UNITED KINGDOM

## Abstract

Despite recombinant protein technology development, proteins isolated from natural sources remain important for structure and activity determination. Ficolins represent a class of proteins that are difficult to isolate. To date, three methods for purifying ficolin-3 from plasma/serum have been proposed, defined by most critical step: (i) hydroxyapatite absorption chromatography (ii) N-acetylated human serum albumin affinity chromatography and (iii) anti-ficolin-3 monoclonal antibody-based affinity chromatography. We present a new protocol for purifying ficolin-3 complexes from human plasma that is based on an exclusive ligand: the O-specific polysaccharide of *Hafnia alvei* PCM 1200 LPS (O-PS 1200). The protocol includes (i) poly(ethylene glycol) precipitation; (ii) yeast and l-fucose incubation, for depletion of mannose-binding lectin; (iii) affinity chromatography using O-PS 1200-Sepharose; (iv) size-exclusion chromatography. Application of this protocol yielded average 2.2 mg of ficolin-3 preparation free of mannose-binding lectin (MBL), ficolin-1 and -2 from 500 ml of plasma. The protein was complexed with MBL-associated serine proteases (MASPs) and was able to activate the complement *in vitro*. In-process monitoring of MBL, ficolins, and total protein content revealed the presence of difficult-to-remove immunoglobulin G, M and A, in some extent in agreement with recent findings suggesting crosstalk between IgG and ficolin-3. We demonstrated that recombinant ficolin-3 interacts with IgG and IgM in a concentration-dependent manner. Although this association does not appear to influence ficolin-3-ligand interactions *in vitro*, it may have numerous consequences *in vivo*. Thus our purification procedure provides Ig-ficolin-3/MASP complexes that might be useful for gaining further insight into the crosstalk and biological activity of ficolin-3.

## Introduction

The complement system is a part of innate immunity and represents the first line of defense against infections. Its activation leads to initiation of the inflammatory response followed by death of the pathogen [1]. There are three pathways of complement activation: classical, alternative, and lectin-based. The lectin pathway in humans is initiated by certain collectins, such as mannose-binding lectin (MBL), collectin-10 (CL-10), collectin-11 (CL-11), and all known ficolins, including ficolin-1 (M-ficolin, p35-related protein), ficolin-2 (L-ficolin, p35) and ficolin-3 (H-ficolin, Hakata antigen, thermolabile β2-macroglycoprotein) [2]. These proteins, in complex with MASPs (MBL-associated serine proteases; MASP-1, MASP-2, and MASP-3), specifically bind to pathogen-associated molecular patterns (PAMPs). Binding of collectins to PAMPs causes substantial conformational changes in MASPs that result in their enzymatic activation. Activated MASPs, in turn, cleave complement components C4 and C2 to generate C3 convertase and subsequently activate downstream cascade components. This may lead to direct lysis of the pathogenic cell or its opsonization, the latter of which facilitates phagocytosis. Furthermore, anaphylatoxins released during complement cleavage are chemoattractants for phagocytes and promote the inflammatory response [3]. Whereas MASP-2 is crucial for the production of C3 convertase, MASP-1 is essential for its activation [4, 5].

Collectins generally (e.g., MBL) and ficolins exhibit some similarities in molecular structure. They are built of multimers (i.e., trimers, tetramers, pentamers, hexamers) of trimeric subunits and contain regions responsible for ligand recognition: a carbohydrate-recognition domain (CRD) in collectins, *e*.*i*. MBL, and a fibrinogen-like (FBG) domain in ficolins. These molecules act as sensors and activators of complement reflecting their particular specificity towards PAMPs.

Ficolins recognize a variety of ligands, including natural complex carbohydrates and monosaccharides; however, they exhibit some specificity towards N-acetylated non-sugar molecules [2, 6–10]. A few reports described the binding of ficolins to bacteria [2, 9–13], fungi [14], viruses [15], parasites [16, 17] and eukaryotic cells [18]. Generally, ficolins differ in their ligand specificity. However there are a few ligands that are common for some of ficolins. Ficolin-1 and -2 recognize d-GlcNAc and all three ficolins (-1, -2, -3) recognize human and bovine acetylated albumin (Ac-HSA and Ac-BSA) [7].

Little is known about the ligands recognized by human ficolin-3. To date no universal structural motif recognized by this lectin was identified, although it is suggested that N-acetyl groups are involved in the binding. Ficolin-3 has been demonstrated to bind to monosaccharides, such as d-Fuc, d-Gal [2, 19], d-GalNAc, Ac-BSA and Ac-HSA [7]. Among natural complex carbohydrate ligands, only the polysaccharide of *Aerococcus viridans* 86965 [11] and O-specific polysaccharides of *Hafnia alvei* PCM (Polish Collection of Microorganisms) 1200 (O-PS 1200), 1203, 1205 and 23 [12], have been identified. O-PS of *H*. *alvei* 1200 LPS used in this study as a ficolin-3 ligand is built of branched pentasaccharide repeating units: →3)-β-d-Qui*p*4NAc-(1→1)-Gro-(3-P→3)-[β-d-Glc*p*NAc3OAc-(1→2)][α-d-Glc*p*-(1→4)]-β-d-Gal*p*-(1→3)-α-d-Glc*p*NAc-(1→. Until recently, ficolin-3 was thought not to recognize any common pathogen, but has subsequently been shown to specifically bind influenza A viruses [15], *Aspergillus fumigatus* conidia [14], *Pasteurella pneumotropica*, enteropathogenic *E*. *coli* (O111ab:H2 serotype) and enteroaggregative *E*. *coli* (O71 serotype) [13]. Ficolin-3 also binds surface antigens of late apoptotic cells [18]. Though the concentration of ficolin-3 in humans (~18 μg/ml) is highest among ficolins and significantly exceeds the level of MBL [20], its role in innate immunity is still not clearly understood. There are sparse reports about its complement-dependent bactericidal (*H*. *alvei*, *P*. *pneumotropica*, *E*. *coli*) [21, 13], antifungal (*A*. *fumigatus*) [14], antiviral (influenza A virus) [15], and antiparasite activity (*Trypanosoma cruzi*, *Giardia intestinalis*) [16, 17].

A detailed investigation of the role of ficolin-3 in innate immunity requires a variety of experimental tools. Common animal models are not applicable, as rodents do not synthesize a ficolin-3 ortholog. Previous *in vitro* studies of the activity and significance of ficolins in the immune system have used serum/plasma-derived or recombinant proteins. However, commercially available recombinant proteins usually differ from natural proteins in ways that could influence their biological properties. For example, N-linked glycosylation of murine ficolin B is essential for the formation of highly oligomeric molecule, which in turn is necessary for association with MASPs and sMAP [22]. In humans, glycosylated ficolin-3 forms oligomers with molecular weights greater than 700 kDa that complex with MASPs and interact with PAMP structures, triggering relevant immune responses. Important in the current context, commercially available recombinant ficolin-3 is devoid of MASPs, making it impossible to examine most biological activities of the native protein associated with complement activation. Additionally, recombinant technology to facilitate purification usually provided proteins with His-tag, which may interfere with some experimental condition. Thus, studies of certain features of the physiological function of ficolins dictate that proteins be obtained from natural resources, for example from human serum or plasma. Moreover proteins isolated from natural sources are invaluable analytical tools for obtaining detailed information about native protein structure, possible modifications, and activity.

Thus plasma-derived preparations are of special interest because, thanks to the association of pattern-recognition molecules with MASP serine proteases, they are not only capable of pattern recognition but are also able to activate MASP substrates. Thus, these preparations may enable investigation of a range of ficolin-3 properties, including specificity, interactions with self and non-self cells, anti-microbial and anti-cancer activity, impacts on complement and coagulation systems, and innate-adaptive immunity crosstalk.

Described value of native ficolin preparations highlights the need for the development of effective protocols for purifying ficolin-3 from plasma/serum. To date, three methods, differing mainly in the column material using in the initial step, have been proposed. These include hydroxyapatite absorption chromatography combined with gel filtration, preparative electrophoresis and affinity chromatography, described by Yae et al. [23], N-acetylated human serum albumin (Ac-HSA)-Sepharose affinity chromatography, used by Zacho et al. [20]; and an antibody-based affinity chromatography approach employing Sepharose 4B-conjugated anti-ficolin-3 monoclonal antibody (mAb) (clone 4H5), reported by Matsushita et al. [24]. Some key limitation of these technologies may be identified. Even though Yae et al. discovered ficolin-3 (“Hakata antigen”), they isolated only very low amounts of monomeric, biologically inactive form of ficolin-3 (by electrophoresis under reductive condition). Regarding Ac-HSA ligand used by Zacho et al. [20], it has been shown to be recognized also by ficolin-1 and ficolin-2 [25, 26]. The latter method based on mAb [23, 24] is highly specific, but may provide a preparation containing both biologically active and inactive ficolin-3. Additionally, any of these methods used natural ligand of ficolin-3 at the affinity chromatography step. Here, we present an alternative affinity chromatography protocol for purification of ficolin-3 complexes from human plasma based on its natural, specific ligand O-PS 1200 isolated from *H*. *alvei* PCM 1200 LPS. The isolated ficolin-3 was in a biologically active complex with MASPs, free of MBL and other ficolins, and thus might be used to test biological effects of ficolin-3-induced activation of the complement lectin pathway. As a result, we have provided biologically active ficolin-3/MASP complexes and information about its specific interaction with immunoglobulins, most likely as an effect of currently investigated cross-talk between PRM and immunoglobulins [27, 28]. As a consequence, the use of such preparation may help to better understand innate immunity mechanisms and generate useful strategies for treatment of complement-related deficiencies and disorders.

## Materials and Methods

### Bacteria and yeasts

*H*. *alvei* strain PCM 1200 was obtained from the Polish Collection of Microorganisms (PCM) at the Institute of Immunology and Experimental Therapy (Wroclaw, Poland). The bacteria were grown in LB medium, killed with 0.5% phenol, and centrifuged using a CEPA flow laboratory centrifuge [12]. Fresh baker’s yeasts (100 g; Lesaffre Polska S.A., Poland) were suspended in 100 ml of demineralized, Milli-Q (mQ)-purified water, washed a few times with 100 ml mQ water by centrifugation for 10 min at 10000 × g and 4°C, autoclaved, and dried (final weight of dried yeast: 22 g) using an ilShin laboratory freeze dryer (The Netherlands).

### Human plasma

Pooled human plasma collected from healthy volunteers was purchased from Biowest SAS (France, cat. no. S4180). Ficolin-3 average concentration was determined as 21.6 μg/ml.

### Preparation of LPS and O-specific polysaccharide

LPS 1200 was extracted from bacterial cells of *H*. *alvei* PCM 1200 by the hot phenol/water method [29] and purified by ultracentrifugation, as previously described [30]. The yields of LPS preparations were approximately 3.0%. Poly- and oligosaccharides were isolated by mild acidic hydrolysis (1.5% acetic acid) at 100°C for 15 min, and fractionated as previously described [31, 32] using Bio-Gel P-10. The yields of O-PS 1200 of *H*. *alvei* PCM 1200 LPS were 17–20%.

### O-PS 1200-Sepharose chromatography

O-PS fraction 1 of *H*. *alvei* PCM 1200 (93.33 mg) was reduced using the NaBH_4_ [33] and purified by size-exclusion chromatography on a column of Bio-Gel P-2 (Bio-Rad, USA), equilibrated with 0.05 M pyridine/acetic acid buffer (pH 5.6). Eluates were monitored using a Knauer differential refractometer (Germany), and the polysaccharide fraction was collected and dried using an ilShin laboratory freeze dryer. The O-PS 1200-Sepharose column was prepared by esterifying reduced O-PS 1200 (76.68 mg) using the 1-ethyl-3-(3-dimethylaminopropyl)carbodiimide method [34] and coupling it to 20 ml CNBr-activated, cross-linked AH (1,6-diaminohexane)-Sepharose CL-4 (Pharmacia Fine Chemicals, USA) [35] via its carboxyl groups (O-PS 1200-Sepharose). A precolumn (20 ml) of CNBr-activated, cross-linked AH-Sepharose CL-4 (Pharmacia Fine Chemicals) was also prepared. The content of free amino groups in AH-Sepharose was determined by reaction with dinitrofluorobenzene, as described elsewhere [36]. Both precolumn and O-PS 1200-Sepharose column were equilibrated with TBS/Ca^2+^ running buffer composed of 10 mM Tris-HCl pH 7.4, 140 mM NaCl, and 5 mM CaCl_2_.

### Purification of Ficolin-3 complexes

Pooled, frozen (-20°C) human plasma (500 ml) was completely thawed in a 37°C water bath, then treated with PEG 6000 (Sigma-Aldrich, USA) as described previously [20], with modifications. Briefly, 1 M CaCl_2_ was added to the collected supernatant to a final concentration of 20 mM and incubated for 1 h at 37°C with stirring followed by overnight incubation at 4°C with stirring. The formed clot was removed by centrifugation for 30 min at 2000 × g and 4°C, and the supernatant was collected. PEG 6000 in TBS (10 mM Tris-HCl pH 7.4, 140 mM NaCl) was added to the supernatant and the purification process was carried as described previously [20]. The supernatant was collected, divided into 10-ml aliquots, and stored at -20°C for separate use in further purification steps. MBL in stored samples was depleted by first mixing aliquots with 200 mg of lyophilized *S*. *cerevisiae* and 50 μl 1 M CaCl_2_ (final concentration, 5 mM) and incubating overnight at 4°C with stirring. Then, the suspension was centrifuged for 20 min at 10000 × g and 4°C, after which CaCl_2_ and l-fucose (Sigma-Aldrich) were added to the supernatant to a final concentration of 5 and 100 mM, respectively. The preparation was incubated 1 h at 4°C with stirring and centrifuged for 20 min at 10000 × g at 4°C. Sepharose-binding molecules were removed by loading the obtained supernatant (10 ml) onto a precolumn connected to the O-PS 1200-Sepharose (flow rate, 1 ml/min for 30 min) and incubated for 30 min at 4°C. Next, the precolumn was detached from the O-PS 1200-Sepharose column and washed separately with 3 column volumes (CV) of TBS/Ca^2+^/Tween (TBS/Ca^2+^, 0.05% Tween 20), 2 CV of eluting buffer (50 mM Tris-HCl pH 7.8, 1 M NaCl, 0.05% Tween 20, 10 mM EDTA), 2 CV of 3 M KSCN, and at least 3 CV of TBS/Ca^2+^/Tween to complete the KSCN elution. The presence of KSCN was determined by reaction with FeCl_3_, which transforms colorless potassium thiocyanate into the deep red iron (III) thiocyanate, Fe(SCN)_3_. The O-PS 1200-Sepharose column was washed with at least 2 CV of TBS/Ca^2+^/Tween to remove all non-bound molecules, monitored by measuring absorbance at 190 and 280 nm, and then washed with 2 CV of 200 mM d-GlcNAc in TBS/Ca^2+^ (pH 7.5) to elute remaining ficolin-1 and ficolin-2 [20, 26, 37, 38]. Finally, ficolin-3 complexes were eluted with 1.5 CV of 1 M sodium acetate (pH 7.5), and the column was flushed with 2 CV of eluting buffer and 2 CV of 3 M KSCN, and equilibrated with TBS/Ca^2+^/Tween. All eluates were collected and concentrated (3000 × g, 4°C) to a final volume of ~500 μl using an Amicon Ultra-15 Centrifugal Filter Unit (MWCO, 30 kDa; Millipore, USA). The concentrated ficolin-3 preparation was further fractionated on a TSKgel G3000SW column (21.5 mm ID × 30 cm, 13 μm; Tosoh Bioscience LLC, Germany), equilibrated with PBS (pH 7.5), under the following conditions: injected volume, 1 ml; flow rate, 5 ml/min; fraction volume, 2.5 ml. The eluate was monitored by measuring absorbance at 280 nm. Each fraction was concentrated or pooled and examined for purity, biological activity, and concentration as described below.

### Dot-blot detection of MBL, ficolins and immunoglobulins

Concentrated fractions (1 μl) were spotted onto nitrocellulose membranes (GE Healthcare, USA). Membranes were blocked with SuperBlock Blocking Buffer (Thermo Scientific, USA) for 1 h and incubated for 1 h with the following primary antibodies: (i) anti-ficolin-3 mouse monoclonal IgG1 (clone 4H5; Hycult Biotech, The Netherlands), (ii) anti-ficolin-1 goat polyclonal IgG (clone G-13; Santa Cruz Biotechnology, USA), (iii) anti-ficolin-2 goat polyclonal IgG (clone G-12; Santa Cruz Biotechnology) or (iv) anti-MBL mouse monoclonal IgG1 (clone HYB 131–01; BioPorto, Denmark), diluted to a final concentration of 0.5 μg/ml in 10 mM Tris-HCl supplemented with 50 mM CaCl_2_ (pH 7.4). Horseradish peroxidase (HRP)-conjugated anti-goat IgG polyclonal rabbit Ab (catalogue number: P0448, Dako, Denmark), anti-mouse IgG monoclonal rabbit Ab (catalogue number: P0260, Dako) and separately used or the mixture of anti-human IgA α chain, IgG γ chain, IgM μ chain polyclonal Ab (the mixture: Dako, catalogue number: P0212; separate Ab: Sigma-Aldrich) were used as secondary antibodies (1 μg/ml, 1 h incubation). Membranes were incubated for 10 min with Immun-Star HRP Chemiluminescent Substrate Kit (Bio-Rad, USA) and visualized using a G:Box Bioimaging System (Syngene, UK). Membranes were washed three times with dot-blot buffer (50 mM Tris-HCl pH 7.4, 0.2 M NaCl) after each step of the procedure. The procedure was carried out at room temperature.

### Ficolin-3, MBL and total protein concentration

Ficolin-3 concentration was measured by ELISA as described previously by Michalski et al. [39], except that microtiter plates were coated with *H*. *alvei* PCM 1200 LPS (5 μg/well) instead of a BSA conjugate of its O-PS fraction. Recombinant human ficolin-3 protein (catalogue number: 2367-FC, R&D Systems, USA) was used as a standard. MBL concentration was determined as described previously [40]. Recombinant human MBL protein (catalogue number: 2307-MB/CF, R&D Systems) was used for calibration curve construction. Total protein concentration was determined using the bicinchoninic acid method (Micro BCA Protein Assay kit; Thermo Scientific) [41].

### Detection of immunoglobulins, thrombin, collectin-11 and C1q in plasma-derived ficolin-3 complexes

Microtitier plate Maxisorp (Nunc) was coated with *H*. *alvei* PCM 1200 LPS (5 μg/well), and then after blocking with 1% BSA in TBS/Ca^2+^, 500 ng of plasma-derived ficolin-3 complexes in MBL-binding buffer (20mM Tris, 1M NaCl, 10mM CaCl_2_, pH 7,4) supplemented with 0,1% BSA was added and incubated overnight at 4°C. After washing in TBS/Ca^2+^, antibodies IgA, IgG and IgM were detected by HRP-conjugated primary antibodies (as above); and ficolin-3, thrombin, C1q and CL-11 were detected by following primary Ig: (i) anti-ficolin-3 mouse monoclonal IgG1 (clone 4H5; Hycult Biotech), (ii) anti- thrombin rabbit polyclonal (catalogue number: ab92621, Abcam, UK), (iii) anti-Complement 1q (C1q) goat polyclonal (catalogue number: 234390, Calbiochem, USA), (iv) anti-collectin-11 (CL-11) rabbit polyclonal (catalogue number: HPA035241, Sigma-Aldrich) and HRP-conjugated secondary antibodies: rabbit anti-goat IgG (catalogue number: P0448, Dako) or goat anti-rabbit IgG antibodies (catalogue number: P0448, Dako) or rabbit anti-mouse IgG (catalogue number: P0260, Dako).

#### Detection of ficolin-3-complexed and free immunoglobulins in plasma-derived ficolin-3 complexes

Ficolin-3 native (active) and heat-inactivated (incubation for 30 min at 56°C) preparations were added (125 ng) to LPS 1200-coated microtitier plate Maxisorp (Nunc). The bound immunoglobulins and ficolin-3 were detected using HRP-conjugated rabbit anti-human Ig G, A and M antibodies (Dako) or anti-ficolin-3 monoclonal antibodies and HRP-conjugated anti-mouse Ig, respectively. The results were compared to active ficolin-3 preparation.

### MASPs detection

Co-purified MASPs were detected by Western blot analysis. Briefly, plasma-derived ficolin-3 complexes (2 μg) were separated on 10% polyacrylamide gels and transferred onto PVDF membranes (Thermo Scientific). After blocking for 2 h with 5% skim milk, membranes were incubated with goat polyclonal anti-MASP-1 (catalogue number: sc-50841, Santa Cruz Biotechnology), rat monoclonal anti-human MASP-2/MAp-19 (Hycult Biotech, catalogue number: HM2191), or goat polyclonal anti-complement MASP-3 (catalogue number: AF1724-SP, R&D Systems) for 2 h at room temperature. The primary antibodies were diluted to a final concentration of 0.5 μg/ml in buffer containing 10 mM Tris-HCl (pH 7.4) and 50 mM CaCl_2_. Next, 1000-fold diluted, HRP-conjugated rabbit anti-goat IgG (catalogue number: P0448, Dako) or anti-rat IgG antibodies (catalogue number: P0450, Dako) were added and the incubation was continued for 1 h at room temperature. After washing, membranes were incubated for 10 min with enhanced chemiluminescence (ECL) substrate (Santa Cruz Biotechnology) and exposed to X-ray film. Membranes were washed six times between each step.

### MASPs activity

The ability of MASPs in plasma-derived ficolin-3/MASP complexes to cleave C3 and C4 was examined according to the method described by Matsushita and Fujita [42]. Briefly, different concentrations of plasma-derived ficolin-3/MASP complexes were incubated with 2 μg of C3 or C4 (Sigma-Aldrich) for 4 h at 37°C. The reactions were terminated by adding SDS-PAGE sample buffer (62.5 mM Tris-HCl, 2.5% SDS, 5% β-mercaptoethanol, 10% glycerol, 0.002% bromophenol blue) and heating at 99°C for 10 min. Proteins were separated on 10% polyacrylamide gels and stained with Coomassie Brilliant Blue to detect cleavage products. Alternatively, MASP-2 activity, based on C4 activation product deposition, was estimated as described previously using plates coated with LPS 1200, C4 from human plasma (catalogue number: C8195, Sigma) and rabbit anti-human C4c antibodies (catalogue number: F0169, Dako) [12]. Conditions excluding activation of complement classical pathway were applied [43]. MASP-1 activity was measured according to a protocol described by Presanis et al. [44] using the MASP-1 fluorescent substrate, Boc-Val-Pro-Arg-aminomethylcoumarin (VPR-AMC) with slight modifications. Briefly, white microtiter Maxisorp plates (Nunc, Denmark) were coated with *H*. *alvei* PCM 1200 LPS (5 μg/well) instead of mannan, which was used in the original protocol for determination of MASP-1 complexed with MBL.

### Surface plasmon resonance (SPR)

SPR studies were performed using a Biacore T200 system (GE Healthcare). Recombinant ficolin-3 (R&D Systems) was immobilized on CM5 sensor chip (GE Healthcare Bio-Science AB) using amine coupling chemistry. Recombinant ficolin-3 was injected as a 50 μg/ml solution in 10 mM sodium acetate (pH 4.0) at a flow rate of 5 μl/min to a level of 15,300 resonance units (RU). The reference flow cell was immobilized with 12000 RU of BSA (Sigma-Aldrich). HBS-N buffer (GE Healthcare) supplemented with Ca^2+^ and Mg^2+^ ions (0.01 M HEPES pH 7.4, 0.15 M NaCl, 0.005% v/v surfactant P20, 5 mM MgCl_2_, 5 mM CaCl_2_) was used as a running buffer and sample buffer. Different concentrations of human IgG, IgA, and IgM (Sigma-Aldrich), recombinant MBL (R&D Systems) and O-PS 1200 were injected over the surface of immobilized recombinant ficolin-3 and BSA (reference flow cell) at a flow rate of 30 μl/min for 150 s or 600 s (O-PS 1200); 0.5% SDS injected for 30 s was used as a regenerator in all SPR experiments. BSA or ethanolamine were used to optimize a reference surfaces for analytes IgM and IgA.

## Results

### Purification of ficolin-3 complexes from human plasma

Ficolin-3 complexes were isolated from human plasma using the following four-stage procedure: (i) PEG 6000 precipitation, as described by Zacho et al. [20]; (ii) yeast and l-fucose incubation, to remove contaminating MBL; (iii) affinity chromatography based on the identified specific ligand, O-PS of *H*. *alvei* 1200 LPS (O-PS 1200); and (iv) size-exclusion high-performance liquid chromatography (SEC-HPLC). Total protein, MBL and ficolin-3 concentration, and the presence of immunoglobulins, ficolin-1, ficolin-2, and MBL contamination (dot-blot) were monitored during the purification procedure (Table 1).

**Table 1:** Ficolin-3 Complexes Recovery and MBL and Total Protein Concentration During the Purification Process.

Purification step	Volume (ml)	Ficolin-3 (mg)	MBL (mg)	Total protein (mg)	MBL (%)	Ficolin-3 recovery^a^ (%)
**Plasma**	500	10.8 (± 0.6)	0.3 (± 0.0)	25616.1 (± 5478.7)	2.5	100.0
**4–9% PEG cut**	100	9.0 (± 0.5)	0.2 (± 0.0)	6140.3 (± 419.0)	1.9	83.3
**Yeast and l-fucose incubation**	100	8.2 (± 0.6)	0.0 (± 0.0)	4689.7 (± 872.1)	0.0	75.3
**Sodium acetate from O-PS 1200-sepharose**	26	6.6 (± 0.7)	0.0 (± 0.0)	4171.2 (± 38.0)	0.0	60.5
**Eluate from G3000SW (fraction 22–25)**	12.2	2.2 (± 1.1)	0.0 (± 0.0)	43.8 (± 11.4)	0.0	19.8
**Eluate from G6000Hr (fraction 22–25)**^**b**^	7.5	1.8 [1.3]	0.0 [0.0]	ND	0.0 [0.0]	18.3 [13.5]

^a^Recovery (%) was determined by dividing the total amount of ficolin-3 at a given purification step by the total amount of ficolin-3 in the starting material (500 ml plasma). Besides G6000Hr eluate, mean average values are presented for 4 measurements. The value in parentheses represents standard deviation.

^b^Single measurement was performed for the G6000Hr eluate, thus ficolin-3 recovery percentage was calculated using plasma ficolin-3 concentration of 19.2 μg/ml (single point measurement–values in square brackets). ND, no data.

**Fig 1:**
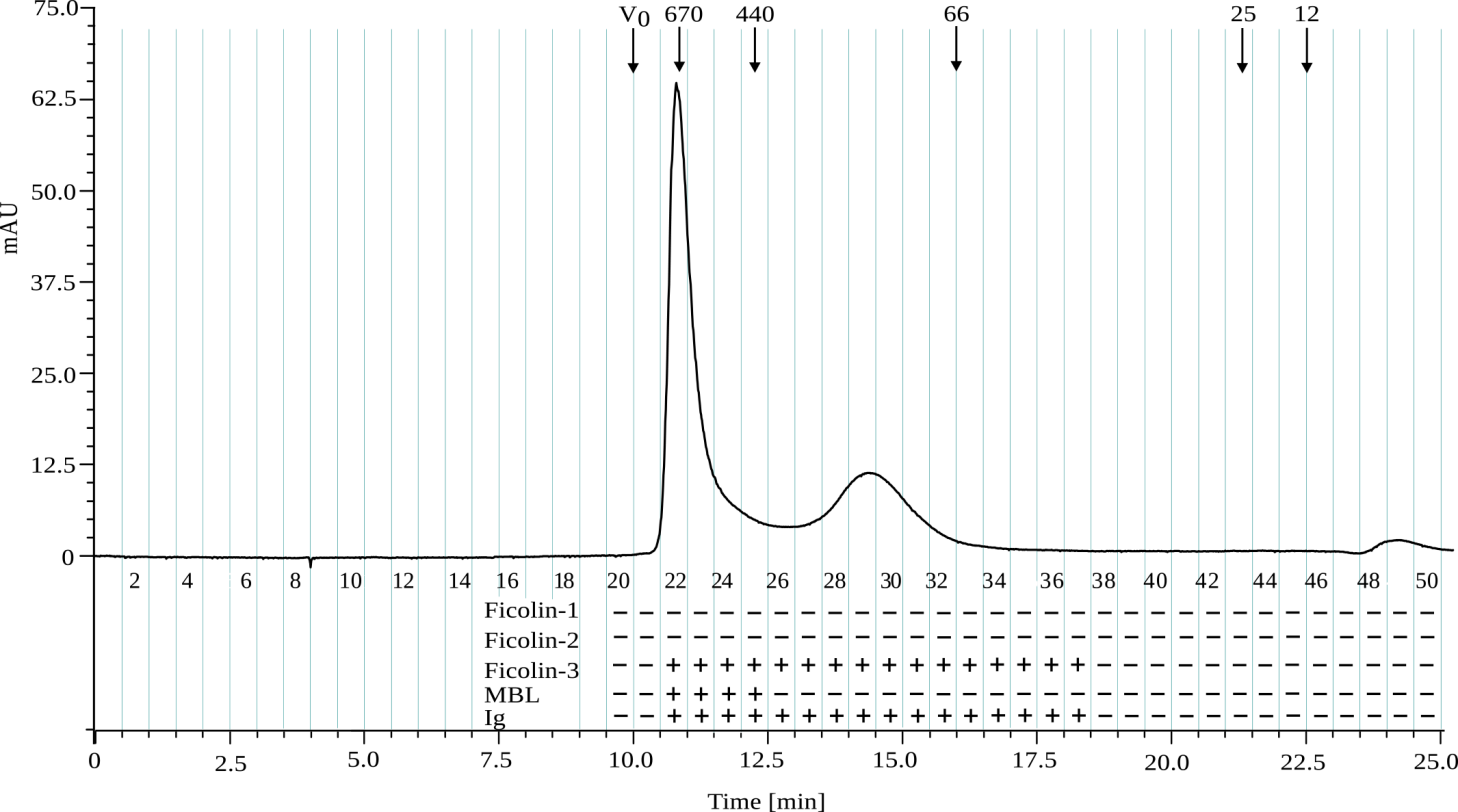
SEC-HPLC chromatography of plasma-derived ficolin-3 complexes on TSKgel G3000SW. Presence of ficolin-1, ficolin-2, ficolin-3, MBL and IgA, IgG, IgM (denoted “Ig”) were detected by dot-blot analysis, where a positive result is indicated by “+”. The apparent molecular mass of thyroglobulin (670 kDa), ferritin (440 kDa), BSA (66 kDa), chymotrypsinogen A (25 kDa) and cytochrome C (12 kDa) was determined in a separate chromatographic run; V_0_, void volume. Even fraction numbers are indicated below the chromatograph line. Absorbance was monitored at 280 nm.

Ficolin-3 concentration was measured by enzyme-linked immunosorbent assay (ELISA) using *H*. *alvei* PCM 1200 LPS as a solid-phase antigen. The plasma (500 ml) was coagulated, after which ficolin-3-containing fraction was precipitated from plasma using a 4–8% PEG cut-off, as described previously [20]. The average recovery of ficolin-3 after precipitation from plasma was 83.3%, whereas MBL contamination was 1.9% (Table 1). At this stage, the total average amount of protein was reduced from 25616.1 mg to 6140.3 mg, of which 9.0 mg was ficolin-3. The resulting preparation was stored in 10-ml aliquots for use in subsequent purification steps. Each 10-ml aliquot was incubated with autoclaved, freeze-dried *S*. *cerevisiae* cells and l-fucose solution, which reduced the MBL contamination [20, 45, 46] from 1.9% to 0.0% (Table 1). At this stage trace amount of MBL was observed only by dot-blot. The concentrated supernatant was purified by affinity chromatography on an O-PS 1200-Sepharose column, resulting in the specific capture of ficolin-3. In this stage, a precolumn of activated AH-Sepharose CL-4 was used to eliminate compounds, including traces of MBL that bound non-specifically to Sepharose [47–49]. The ficolin-3-containing fraction was eluted with 1 M sodium acetate, yielding a total recovery of 60.5%. The final fraction obtained from all aliquots contained 6.6 mg (mean average) of ficolin-3 (Table 1). Ficolin-3 complexes were further purified by SEC-HPLC on a TSKgel G3000SW silica-based column (Fig 1), which separated ficolin-3 high-molecular-weight oligomers (>440 kDa, fractions 22–25) from low-molecular-weight oligomers (66–440 kDa, fractions 26–32), and low-molecular-weight compounds and salts (<12 kDa, fractions 48–50). At this stage, the total average amount of protein per 500 ml was still 43.8 mg, of which 2.2 mg was ficolin-3. However it must be noted that two different methods were used for ficolin-3 (LPS 1200-based ELISA, recombinant ficolin-3 was used for calibration curve) and total protein measurements (the bicinchoninic acid method). The final ficolin-3 average recovery calculated for 500 ml of plasma was 19.8%. As an alternative final step, SEC-HPLC on divinylbenzene-based, polystyrene cross-linked column TSK G6000HR (S1 Fig) was used instead of G3000SW to minimize the significant loss of ficolin-3 on silica-based resins [50]. G6000HR-based separation was performed once and obtained results were compared with results for only one set of measured samples (Table 1, values in square brackets). However, this alternative achieved only a slight improvement, producing a final ficolin-3 recovery of 18.3% (in comparison to 13.5% for single measurement of G3000SW eluate), but it allowed increasing the amount of ficolin-3 by 35.6%.

**Fig 2:**
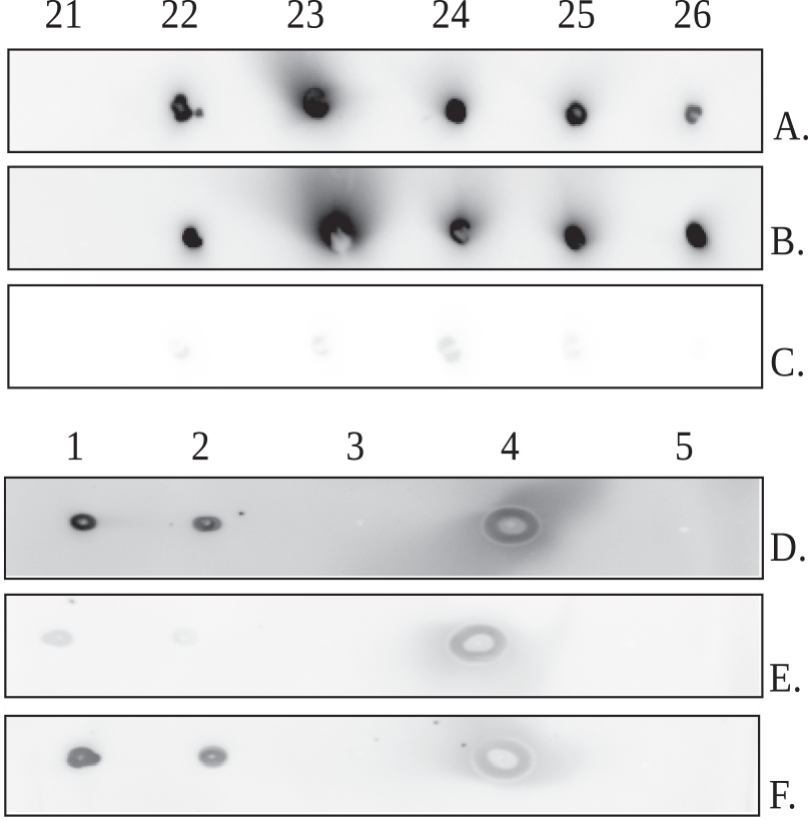
IgA, IgG, IgM, and MBL tracking in SEC-HPLC fractions. Dot-blot analyses were performed using (A) anti-ficolin-3 mAb; (B) anti-IgA α chain, IgG γ chain, IgM μ chain polyclonal Ab; and (C) anti-MBL mAb. Fractions 22–25 were eluted from the TSKgel G3000SW column (Fig 1) and concentrated 9-fold. IgA (D), IgG (E), and IgM (F) tracking in ficolin-3 complexes with dot-blotting. Samples: 1. Ficolin-3 preparation, concentrated eluate from G3000SW column (fractions 22–25); 2. 10-fold diluted ficolin-3 preparation; 3. 100-fold diluted ficolin-3 preparation; 4. Human plasma; 5. Recombinant ficolin-3.

To identify fractions of interest and potential contaminants (see total protein concentration in Table 1), we examined fractions 20–37 for the presence of ficolin-1, ficolin-2, ficolin-3, MBL, IgA, IgG, and IgM by dot-blot assay (Fig 1). Dot-blot analyses of fractions 21–26 for ficolin-3 (Fig 2A), immunoglobulins (Fig 2B) and MBL (Fig 2C) showed a significant amount of Ig and trace amounts of MBL, a result in agreement with data for pooled fractions 22–25 (Table 1). Since preliminary monitoring of Ig contamination was performed with the mixture of anti-IgA α chain, IgG γ chain, IgM μ chain polyclonal antibodies, the next step involved the use of separate antibodies to determine the class of Ig in the ficolin-3 preparation (Fig 2D–2F). Dot-blot results indicated the presence of each class of antibodies, IgG, IgA and IgM, however unexpectedly stronger reactivity was observed with anti-IgA α chain and -IgM μ chain polyclonal antibodies, suggesting the hindered access of detecting antibodies to IgG epitopes due to the involvement of IgG γ chain in interaction with ficolin-3 reported previously by Panda et al. [27, 28] or differences in detection antibodies affinity.

**Fig 3:**
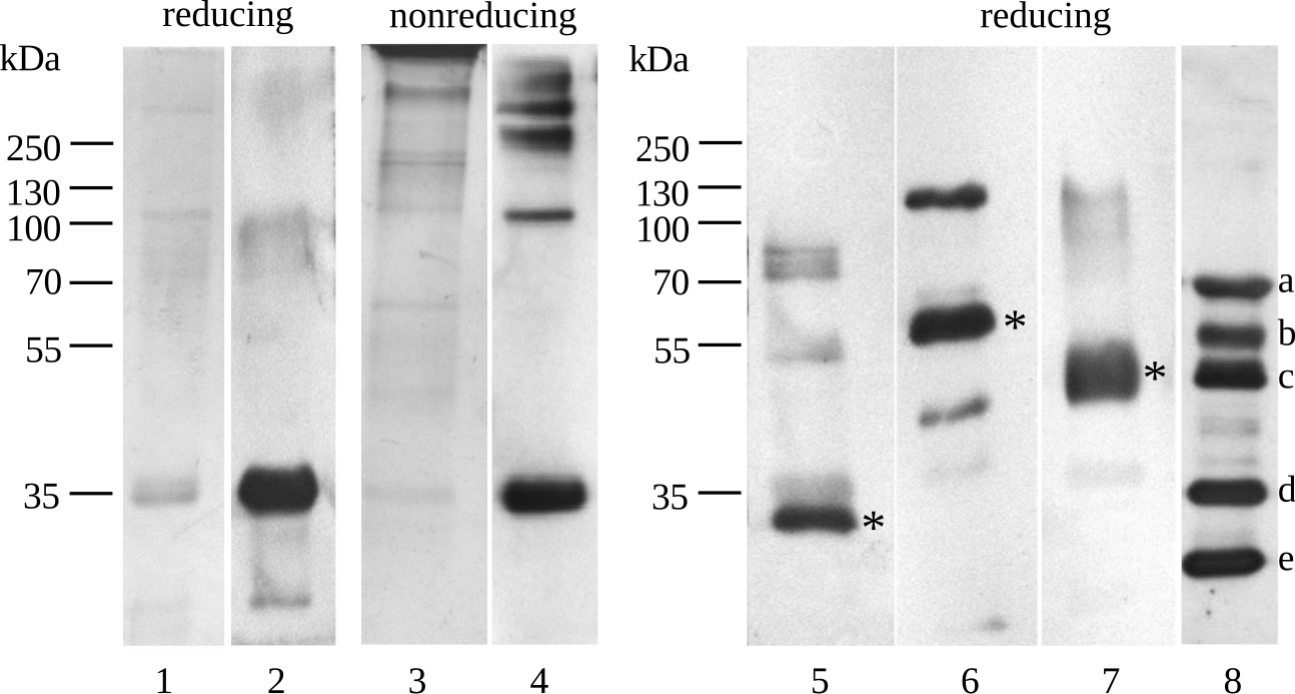
Detection of ficolin-3, MASPs and Ig in plasma-derived ficolin-3 complexes. Analysis of ficolin-3 preparation (1 μg of final product) fractionated on a 10% polyacrylamide gel under reducing (lanes 1, 2, 5–8) or non-reducing (lanes 3–4) conditions. Coomassie Blue stain (lanes 1, 3) and Western blotting using anti-ficolin-3 (lanes 2, 4), anti-MASP-1 (lane 5), anti-MASP-2 (lane 6), and anti-MASP-3 (lane 7) antibodies. The last lane (8) is the strip 4 re-probed with anti-human IgG, IgA and IgM. MASP bands are marked with asterisks (*). A, b, c–heavy chains of IgM (a), IgA (b), IgG (c); d- ficolin-3 monomer; e- light chains of Ig. The positions of the molecular mass markers are indicated in kDa.

In summary, our purification procedure yielded an MBL- and ficolin-1/2-free preparation of ficolin-3 complexes (average mean 2.2 mg) from 500 ml human plasma with a final recovery of 19.8% (Table 1). SDS-PAGE analyses under non-reducing conditions revealed the presence of high-molecular-weight oligomers (Fig 3, lines 3 and 4) as active complexes with MASPs (Fig 3, lines 5–7). The fractionation under reducing conditions showed a prominent band with a molecular weight of 28 kDa (Fig 3, lane 5) corresponded to the MASP-1 B chain, whereas lighter bands above probably correspond to the unreduced chains. A MASP-2 band (Fig 3, lane 6) was observed at a molecular weight greater than 55 kDa; MASP-3 was also detected (Fig 3, lane 7).

#### Detection of immunoglobulins, thrombin, collectin-11 and C1q in plasma-derived ficolin-3 complexes

Although, trace contaminants were observed by SDS-PAGE analysis of plasma-derived ficolin-3 complexes (Fig 3, lines 1 and 3), the content of the preparation was checked for Ig presence after PAGE fractionation under reducing conditions. The strip 4 was re-probed with anti-human IgG, IgA and IgM antibodies showing the presence of heavy and light chains of Ig (Fig 3, line 8). Contrary to dot-blot results obtained for ficolin-3 complexes, all bands revealed similar intensity. Further, the preparation was checked for the presence of possible contaminants or complexed molecules that may affect binding and biological activity of the preparation (S3 Fig). It was shown by interaction of LPS 1200 with the final preparation via ficolin-3, followed by detection of thrombin (capable to cleave VPR-AMC), C1q (classical pathway), CL-11 (LP), together with IgA, IgG, IgM. Performed ELISA confirmed the presence of Ig and excluded the presence of other molecules. It showed also that binding between most of detection IgG (besides anti-IgA antibody) and LPS 1200 might be neglected. To show that most of immunoglobulins were present as complexes with ficolin-3, additional ELISA was performed, which was based on interaction between ficolin-3 and LPS 1200 (S4 Fig). Two types of final preparations were used, native and heat-inactivated, followed by ficolin-3 and total Ig content measurement. The thermal inactivation of ficolin-3 preparation was associated with the decline of ficolin-3 deposition on LPS 1200 as well as with clear reduction of Ig binding. It may indicate that majority of Ig was present as a part of ficolin-3 complexes and suggests that majority of Ig complexed with ficolin-3 binds to LPS via ficolin-3 molecule.

### Biological activity of plasma-derived ficolin-3 complexes

**Fig 4:**
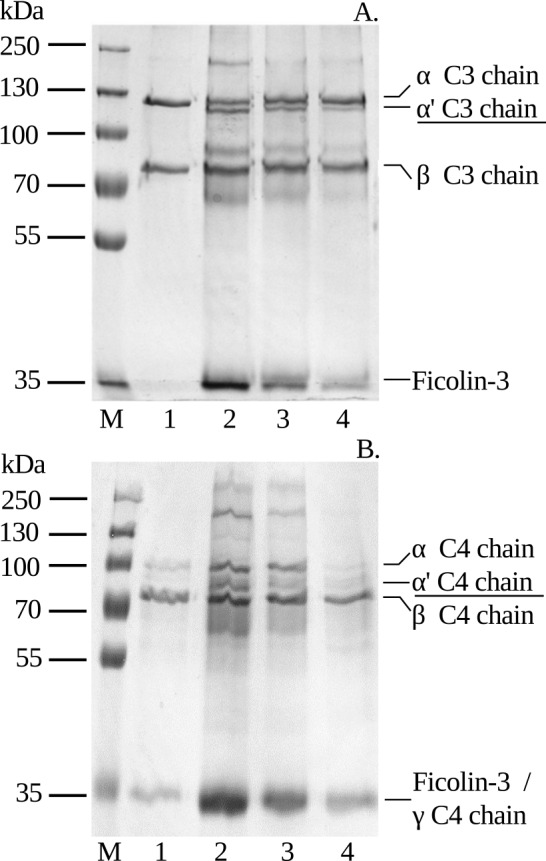
Cleavage of complement components C3 and C4 by plasma-derived ficolin-3/MASP complexes. C3 (A) or C4 (B) was incubated with different concentrations of ficolin-3 preparation: 5 μg (lane 2), 2.5 μg (lane 3), 1.25 μg (lane 4), and control sample without ficolin-3 (lane 1). The cleavage of C3 and C4 was examined by SDS–PAGE and Coomassie Blue staining. M, molecular mass markers. Description of cleavage products is underlined.

To determine whether plasma-derived ficolin-3 is complexed with active MASPs, we incubated different concentrations of the preparation with C3 or C4 and examined reaction products by SDS-PAGE with Coomassie Blue staining (Fig 4). Both complement components were cleaved in a concentration-dependent manner, yielding C3α’ (Fig 4A, lines 2–4) and C4α’ chains (Fig 4B, lines 2–4).

**Fig 5:**
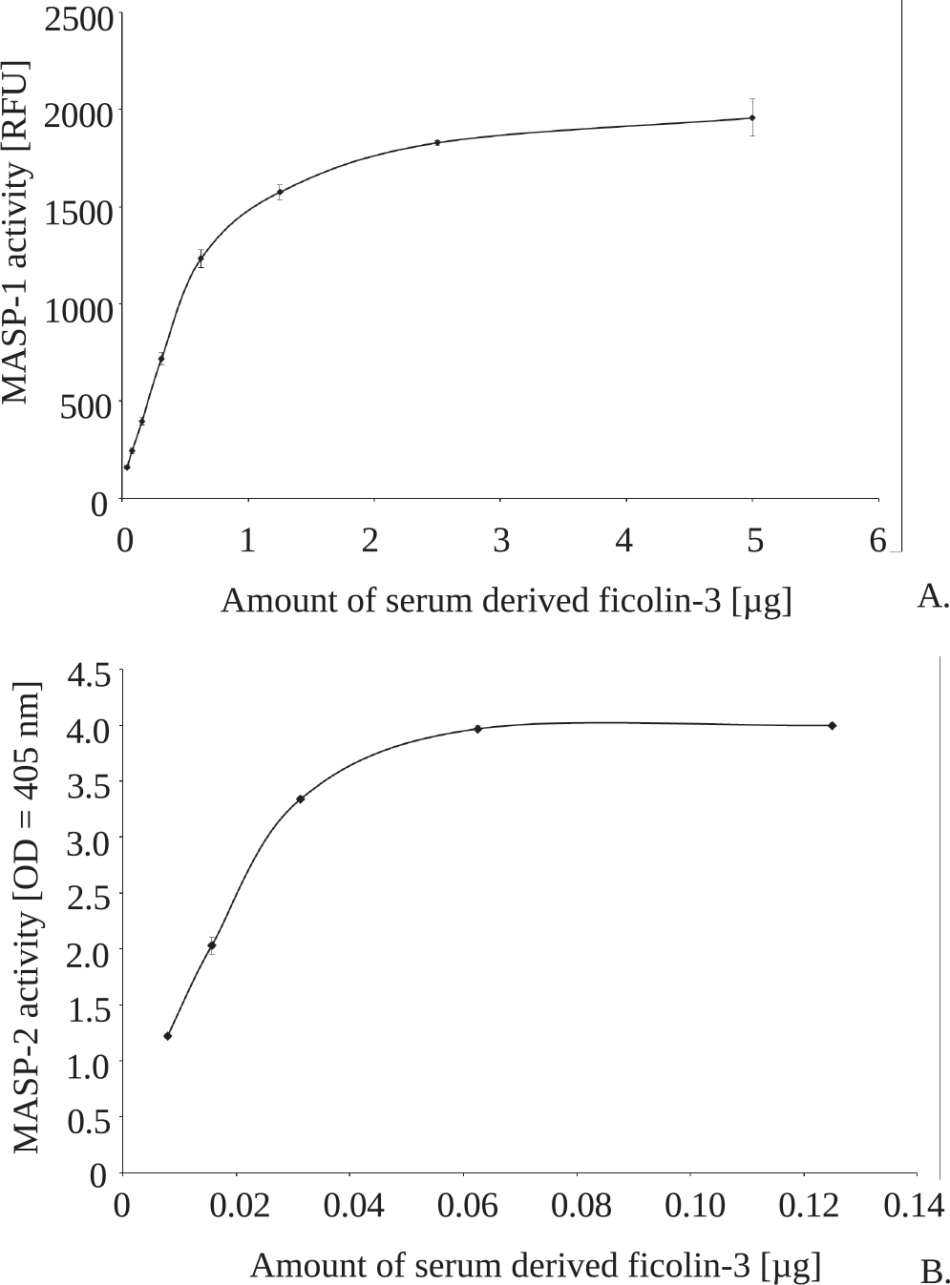
Concentration-dependent activity of plasma-derived ficolin-3/MASP-1 and ficolin-3/MASP-2 complexes to activate LP. (A) MASP-1 activity is expressed as the fluorescence of a product emerging due to the capacity of MASP-1 to cleave VPR-AMC substrate. (B) MASP-2 activity was determined by detection of C4 activation products deposition assay by specific antibodies. Conditions excluding activation of complement classical pathway were applied (Petersen et al., 2001).

The activity of ficolin-3/MASP-1 complexes was examined using the synthetic substrate, Val-Pro-Arg-aminomethylcoumarin (VPR-AMC) and LPS 1200 as a solid-phase ligand for ficolin-3, and was expressed as the fluorescence of cleaved VPR-AMC (Fig 5A).

Ficolin-3/MASP-2 activity was demonstrated using a complement C4 activation products deposition assay (Fig 5B), which showed that plasma-derived ficolin-3 complexes exhibited concentration-dependent activity to cleave C4. C4 degradation products were detected by polyclonal primary rabbit anti-human C4c antibodies and secondary goat anti-rabbit IgG antibodies conjugated with HRP. Similarly, LPS 1200 was used as a solid-phase ligand for ficolin-3.

### Interactions between recombinant ficolin-3 and immunoglobulins

**Fig 6:**
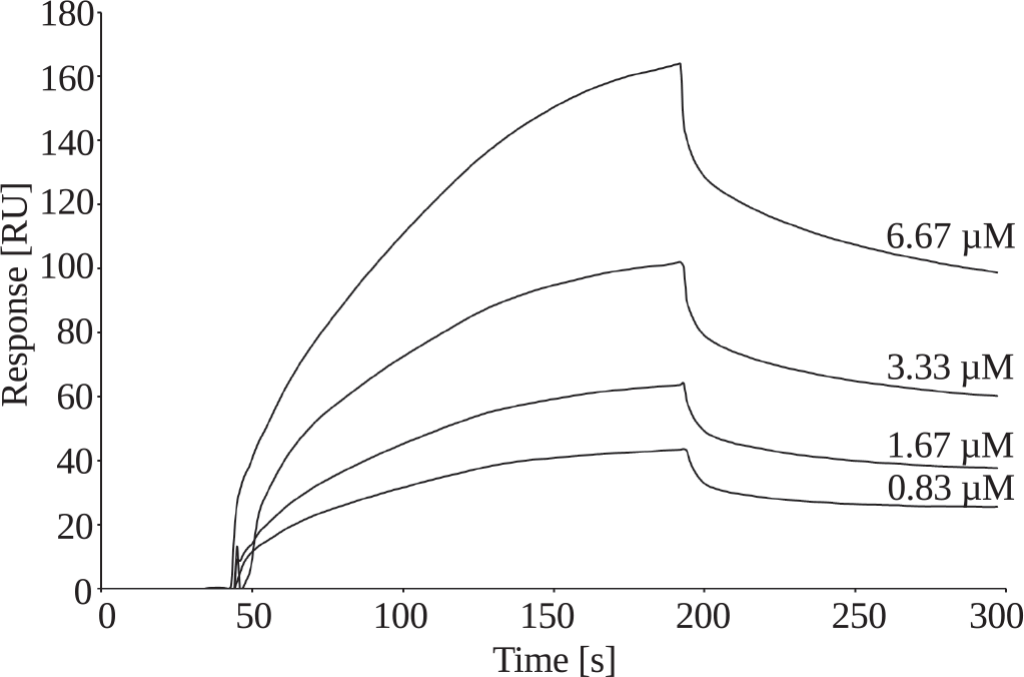
SPR analysis of IgG binding to recombinant ficolin-3. Ficolin-3 was immobilized on a CM5 chip. Ficolin-3 concentrations are indicated adjacent to sensorgrams. RU, resonance units.

In-process monitoring of ficolin-3 purification demonstrated the difficulty in eliminating contaminating IgA, IgG, and IgM from plasma-derived ficolin-3 preparations (Figs 1 and 2B, Table 1). Since Panda et. al previously showed that natural IgG and ficolin-3 interact [27, 28], we investigated binding between recombinant ficolin-3 and human IgG (Fig 6), IgA and IgM (S2 Fig) using surface plasmon resonance (SPR). Additionally, recombinant MBL was also analysed, since it was also observed as the contamination during preliminary steps of the purification process. Ficolin-3 (ligand) was immobilized on a CM5 chip, and different concentrations of Ig in buffer supplemented with Ca^2+^ and Mg^2+^ ions were injected over the chip surface. Human IgG exhibited concentration-dependent binding to ficolin-3 (Fig 6).

**Fig 7:**
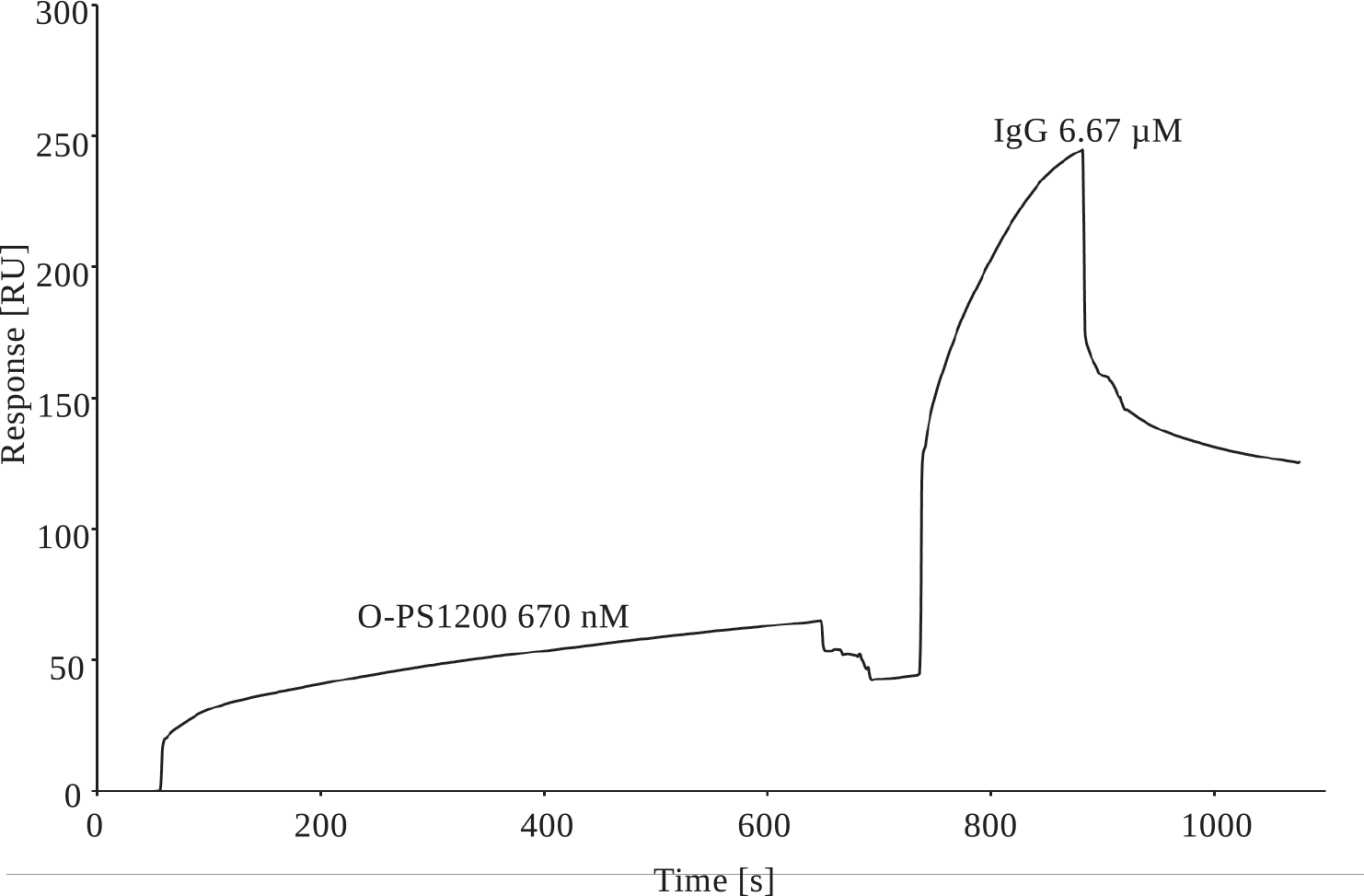
SPR-based competition assay of recombinant ficolin-3 interaction with O-PS 1200 and IgG. Two-step SPR analysis of the interaction of immobilized recombinant ficolin-3 with 670 μM O-PS 1200 and 6.67 μM human IgG. No regeneration step was included between O-PS and IgG. Sensor chip: CM5. RU, resonance units.

In contrast, no binding was observed between recombinant ficolin-3 and recombinant human MBL, used as an analyte and negative control (S2 Fig). The moderate binding was observed for IgM for reference surface (reference flow cell) with immobilized ethanolamine. No interpretable data were obtained for IgA, due to the difficulties with reference cell optimization. Both ethanolamine and BSA showed strong binding with IgA. Moreover BSA used as a reference surface showed strong interactions with IgM. Reference cell optimization experiences with BSA may indicate non-specific interactions between BSA and IgA and IgM. The specificity of the interaction between ficolin-3 and IgG was further examined using a competitive binding assay in which 670 nM O-PS 1200 was first injected over ficolin-3, followed by addition of human IgG. These assays revealed simultaneous binding of both analytes to ficolin-3 (Fig 7), suggesting the presence of different binding sites for each analyte.

## Discussion

To provide active ficolin-3 complexes for further investigation of the ficolin-3 role in innate immunity, we here developed an affinity chromatography method for purification of native ficolin-3 complexes from human plasma and used the resulting protein to assess complement activation by ficolin-3/MASP complexes. The key distinguishing features of our method for isolation of native ficolin-3 complexes from plasma are: (i) an affinity chromatography step using LPS-derived O-PS 1200, a specific ligand for ficolin-3 from *H*. *alvei* PCM 1200 that we have previously shown is effective for measuring ficolin-3 concentration and activity [12, 21, 39, 51–54] and (ii) yeast and l-Fuc incubation step to eliminate MBL. The O-PS 1200 ligand exclusively interacted with ficolin-3 and showed no binding with ficolin-1, ficolin-2 and MBL and relatively weak with Ig (S3 and S4 Figs). Contrary to other methods, we have presented here for the first time data for MBL, ficolins and Ig monitoring in plasma-derived ficolin-3 preparation. The protocol presented here enables the recovery of a preparation of active, oligomerized ficolin-3/MASP complexes practically free of MBL, ficolin-1, and ficolin-2. Moreover, our method requires no hazardous substances or extreme pH values to elute Sepharose-bound ficolin-3 complexes, thereby limiting the risk of structural changes and biological inactivation. We found that ficolin-3/MASP complexes were able to cleave not only C4, but also C3 directly, as was demonstrated previously [55]. Such a phenomenon has previously been reported for MBL/MASP complexes and is suspected to contribute to complement activation via the alternative pathway [42]. All observed binding activities were dependent on LP, since thrombin (capable to cleave VPR-AMC, similarly to MASP-1) was not detected in the complex and C1q (classical pathway) and CL-11 (LP) content might be neglected (S3 Fig). Additionally, MASP-2 dependent activities were measured in conditions excluding activation of complement classical pathway (Petersen et al., 2001).

Contrary to other methods involving PEG precipitation and/or HPLC, we included the incubation step with yeast and l-fucose that was chosen because yeast mannans and l-fucose were previously identified as MBL ligands [44, 46, 56–58]. The step has been proved very effective in reducing MBL and most probably other collectins contamination (Table 1). The trace amount of MBL detected in pooled HPLC fractions containing ficolin-3/MASPs complexes are likely irrelevant for most biological experiments, such as identification of a universal structural motif recognized by ficolin-3 by SPR or saturation transfer difference spectroscopy (STD)-NMR, or investigation of ficolin-3/MASP complex-dependent complement–coagulation crosstalk.

Regarding HPLC step, we observed significant losses of ficolin-3 during G3000SW-based fractionation, reflecting the affinity of ficolins for silica gels. It is known that ficolin-2 binds to silica [50], as manifested by the significantly lower concentration of ficolin-2 in sera prepared from tubes containing silica as a clot activator compared with other serum preparations. This might not be the case for ficolin-3, since only negligible variation was observed for different serum samples [50]. Nevertheless, we decided to test the performance of a TSK G6000HR column. Significant improvement was observed, although additional optimization of chromatography medium might further improve the yield.

The methodology we propose offers some advantages over previously developed protocols. Most importantly, it utilizes a highly specific ligand for ficolin-3, foreclosing cross-reactivity with ficolin-1, ficolin-2 and MBL. By contrast, the Ac-HSA ligand used by Zacho et al. [20] has been shown to interact with ficolin-1 and ficolin-2 [25, 26]. The previously reported mAb-based isolation method [23, 24] is highly specific, but may provide preparation containing both biologically active and inactive ficolin-3. Additionally, the polysaccharide ligand used in our purification process shows enhanced thermal stability, providing increased stability of the chromatography medium and allowing for effective column regeneration; moreover, it might be possible to obtain this ligand in a synthetic form in future. Regarding isolation efficiency, proposed method provided approx. 2.2 mg from 500 ml of plasma. It represents relatively high yield in comparison with hydroxyapatite absorption chromatography– 0,016 mg of pure monomeric ficolin-3 devoid of MASP/500 ml [23] and is comparable with NAc-HSA-Sepharose affinity chromatography– 1.15 mg/500 ml [20]. It should be noted that hydroxyapatite absorption chromatography was developed for physicochemical characterisation of the protein, not efficient isolation. No efficiency information was provided by Matsushita et al. [24].

To further validate our protocol, we included in-process monitoring of MBL, other ficolins, total proteins, and Ig. Despite great efforts to reduce contamination, other proteins besides ficolin-3, prominently including IgG, still contributed significantly to the total protein in our samples (Table 1). However, it is possible that a certain amount of “other protein” detected in samples may be biologically inactive ficolin-3 that is not detected by our LPS 1200-based ELISA. Additional efforts to purify active ficolin-3/MASPs complexes free from IgA, IgM and IgG were unsuccessful, highlighting the possibility of natural affinity of immunoglobulins for ficolin-3. These findings are in accord with the cooperation between ficolin-3 and human IgG reported by Panda et al., especially in infection-inflammation-like conditions (i.e., lower pH and calcium concentration) [27, 28]. Panda et al. demonstrated binding of human serum-derived natural IgG to ficolin-3 immobilized on a GlcNAc-BSA-covered CM5 chip. They have demonstrated an interaction between immobilized IgG and the recombinant FBG domain of ficolin-3. Moreover, they reported pH-dependent (normal and inflammation conditions) dissociation constants K_D_ for ficolin-3 FBG domain and IgG interactions within the range 10^−7^–10^−9^ [27], suggesting a strong binding between molecules. In addition, Lei et al. recently reported the cooperation of IgM and ficolin-3 in activating a complement attack on cancer cells [59]. Le et al. reported similar issues with immunoglobulin contamination during purification of ficolin-like proteins on a GlcNAc-Sepharose column [47]. Their findings indicated cooperation between innate and adaptive immunity. It was shown for the final preparation that majority of Ig is present in complexes with ficolin-3 and are detectable on the surface of ficolin-3 bound to LPS 1200 in ELISA conditions (S4 Fig). Our results clearly demonstrated weaker detection of Ig bound to LPS 1200 after ficolin-3 thermal-inactivation. SPR analyses presented in the current study clearly showed that recombinant ficolin-3 (immobilized ligand) interacts with IgG in a concentration-dependent manner (Fig 6). SPR analysis indicated also the binding between ficolin-3 and IgM. No interpretable data were obtained for IgA, due to the difficulties with reference cell optimization. Contrary to IgG, IgM and IgA interacted also with BSA used as a reference surface, what may suggest non-specific binding of these molecules and the need to explore this issue with the use of other analytical techniques. Thus the presence of IgM and IgA in obtained ficolin-3 preparation might be also a result of the crosstalk. In this context, it is interesting that unexpectedly IgG showed in dot-blot analysis (Fig 2D–2F) and ELISA (S3 Fig) much more weaker reactivity than IgA and comparable reactivity with IgM., what was in contradiction with our SPR result. Affinity of detection antibodies might be one reason for dot-blot results intensity. However, considering results published by Panda et al., one reason for such results is specific interaction between plasma-derived ficolin-3 with IgG *via* Fc region of IgG. As a consequence Fc region (γ chain) of IgG in the preparation is not accessible for anti-IgG γ chain antibody used for the detection. Moreover, using competition assays with O-PS 1200 and IgG, we demonstrated that ficolin-3 was able to simultaneously bind both IgG and O-PS 1200 (Fig 7), suggesting the presence of different binding sites for these ligands. It is known that ficolin-3 ligands identified to date bind at a single site [45]. Panda et al. discovered a new binding site in ficolin-3 that is exposed upon interaction with natural IgG [28]. Discovered binding was the strongest under reduced levels of calcium and pH (pH 6.5, 2.0 mM calcium), conditions that are somewhat different than those used here (pH 7.4, 5 mM calcium).

In summary, the purification procedure presented here provides natural IgG-ficolin-3/MASP complexes that might be useful for gaining further insight into the biological activity and specificity of ficolin-3. The obtained ficolin-3 preparation contained also IgA and IgM, but specificity of their interactions with the ficolin-3 have to be further explored. Although IgG-ficolin-3 binding does not appear to influence ficolin-3-ligand interactions via the FBG domain *in vitro*, it may have numerous consequences *in vivo*. Contrary to other purification methods we showed that ficolin-3 purification was a big challenge due to the crosstalk between pattern-recognition molecules and immunoglobulins, a hot phenomenon described recently by a few research group within the field [27, 28]. Moreover described difficulties and observations were not included in previous methods descriptions, what makes our observation important for further studies on ficolin-3 isolation, specificity and activity.

## Supporting Information

S1 Fig SPR analysis of human IgG, IgM and recombinant MBL binding to recombinant ficolin-3. Ficolin-3 was immobilized on a CM5 chip and IgG, IgM and MBL were used as analytes. Analytes concentrations are indicated adjacent to sensorgrams. RU, resonance units.
(TIFF)

S2 Fig SEC-HPLC chromatography of plasma-derived ficolin-3 complexes on G6000Hr column. Absorbance was monitored at 280 nm.
(TIFF)

S3 Fig Detection of immunoglobulins, thrombin, C1q and CL-11 in plasma-derived ficolin-3 complexes. Microtiter plate was coated by LPS 1200 and 500 ng of plasma-derived ficolin-3 complexes were added, and followed by ficolin-3, IgA, IgG, IgM, thrombin, C1q and CL-11 detection (grey bars). In case of control, the addition of plasma-derived ficolin-3 complexes (white bars) was omitted.
(EPS)

S4 Fig Detection of ficolin-3-complexed and free immunoglobulins in plasma-derived ficolin-3 complexes. The presence of immunoglobulins bound to ficolin-3 interacting with LPS 1200 was detected for native (grey bars) and heat-inactivated (56°C) (white bars) ficolin-3 preparation.
(EPS)
