# Supplementary figures and images for: A New Ligand-Based Method for Purifying Active Human Plasma-Derived Ficolin-3 Complexes Supports the Phenomenon of Crosstalk between Pattern-Recognition Molecules and Immunoglobulins
Source: PLoS One. 2016 May 27;11(5):e0156691. doi: 10.1371/journal.pone.0156691 (PMC4883783; doi:10.1371/journal.pone.0156691)

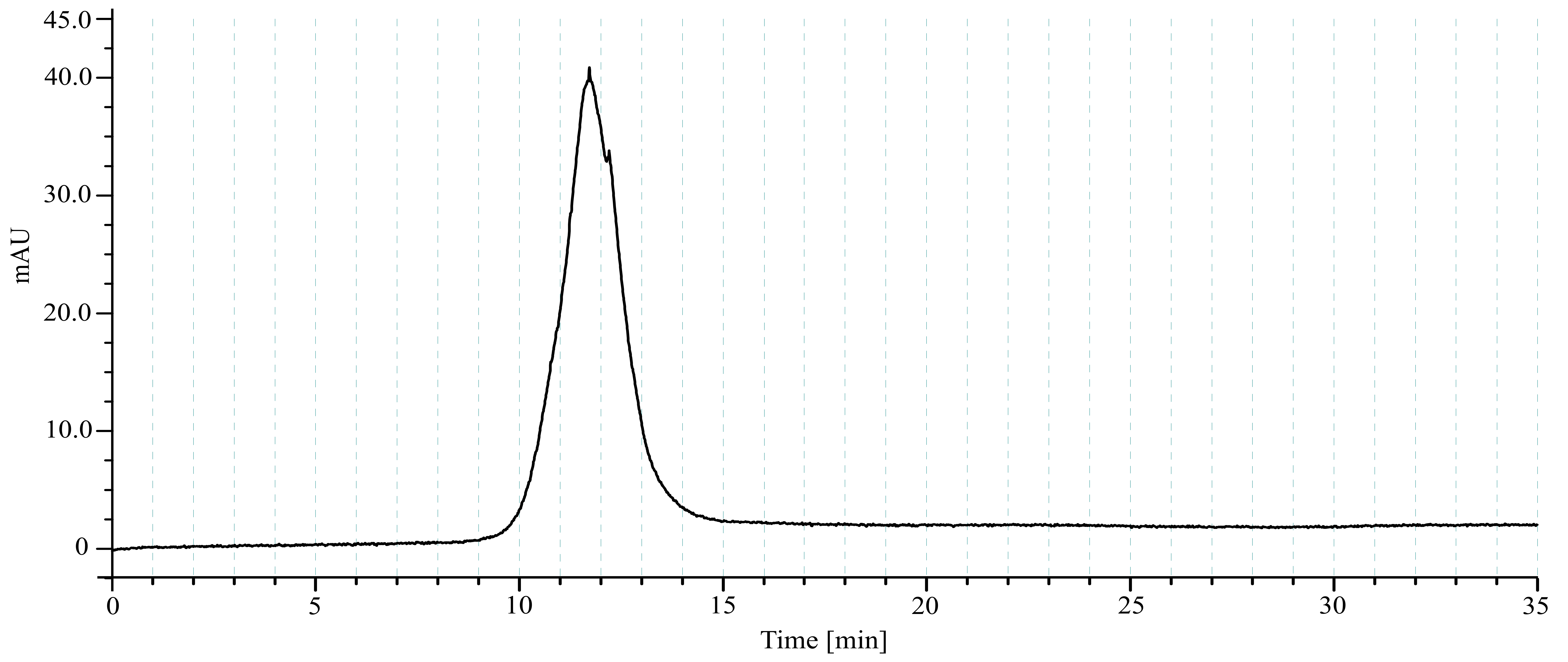

Supplement: S1 Fig — Ficolin-3 was immobilized on a CM5 chip and IgG, IgM and MBL were used as analytes. Analytes concentrations are indicated adjacent to sensorgrams. RU, resonance units. (TIFF) [file pone.0156691.s001.tiff]

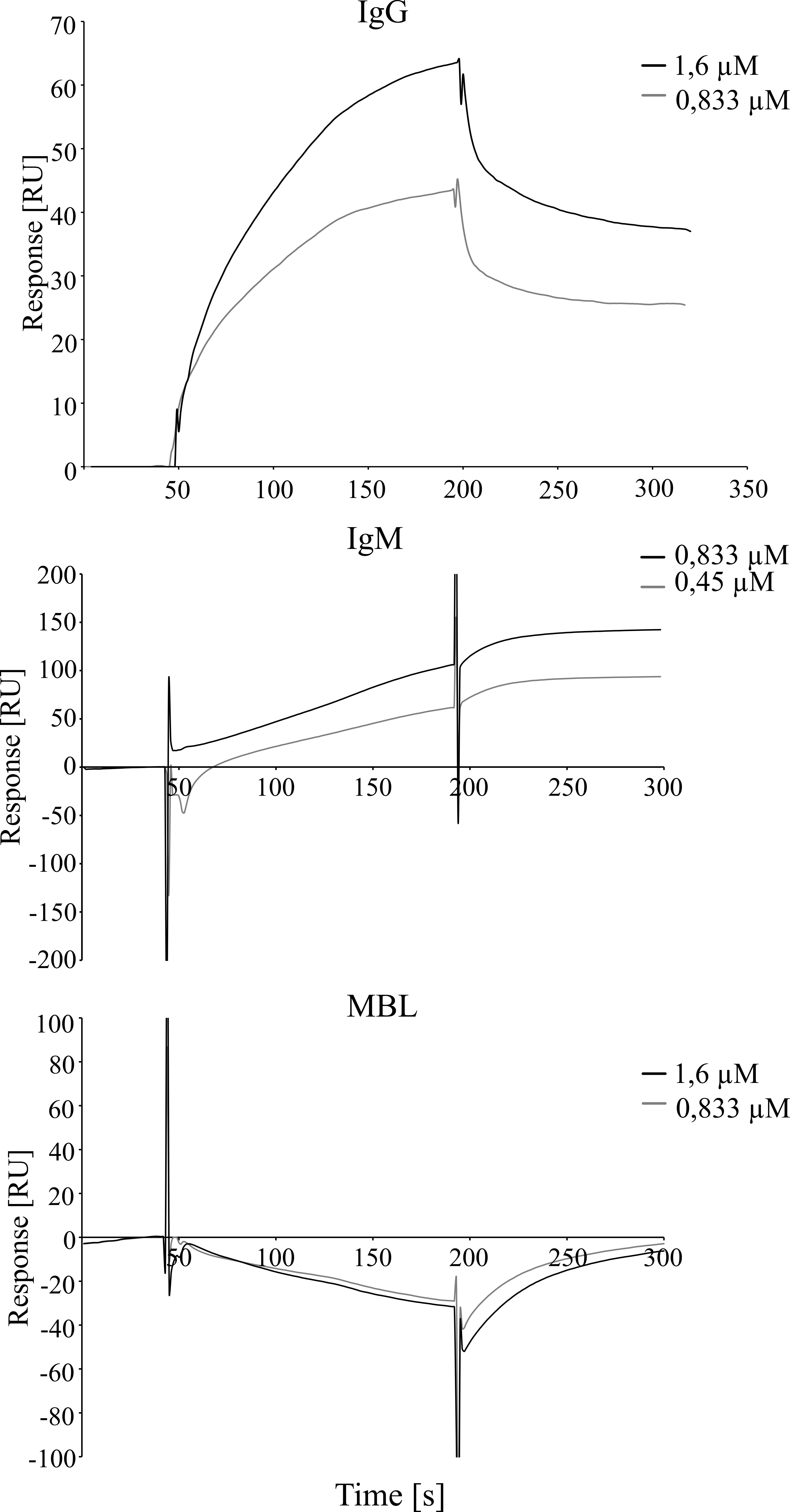

Supplement: S2 Fig — Absorbance was monitored at 280 nm. (TIFF) [file pone.0156691.s002.tiff]
